# Temporal Changes in Species, Phylogenetic, and Functional Diversity of Temperate Tree Communities: Insights From Assembly Patterns

**DOI:** 10.3389/fpls.2019.00294

**Published:** 2019-03-19

**Authors:** Jung-Hwa Chun, Chang-Bae Lee

**Affiliations:** ^1^Research Planning and Coordination Division, National Institute of Forest Science, Seoul, South Korea; ^2^Department of Forestry, Environment and Systems, Kookmin University, Seoul, South Korea

**Keywords:** community assembly, deterministic process, forest strata, functional diversity, neutral process, phylogenetic signal, species diversity, phylogenetic diversity

## Abstract

Species-based approaches to the analysis of changes in successional community assemblages are limited in the ability to reflect long-term evolutionary and functional trait responses of organisms to environment change. Recent advances in concepts and analyses of community phylogenetics and functional traits have improved the interpretation and understanding of community assembly processes. Here, we examined phylogenetic signals of four functional traits such as maximum height, leaf size, seed mass and wood density in woody plant species and temporal changes in species, phylogenetic, and functional diversity among forest strata (i.e., whole, overstory, and understory strata) at four forest long term ecological research sites in South Korea. A census of woody plant species was implemented in a 1-ha permanent plot of each study site every 5 years. We analyzed community structure and compositional turnover using twenty-five 20 × 20 m^2^ quadrat data converted from 1-ha plot data of each site. We found that phylogenetic signals for four functional traits were low but significant, indicating that phylogenetic diversity may be used as a crude surrogate measure of functional diversity. Temporal changes in alpha and beta components of the three diversity differed among forest strata and four study sites over time. This study also revealed that the temporal changes of phylogenetic and functional diversity for understory strata in a forest, which were consecutively damaged by typhoon, were more extreme and larger than those of understory strata in the other sites. Therefore, our study supports recent studies that plant community structures differ among forest strata and such differences of community structure among sites can be accelerated by disturbance. Although the role and relative importance of niche-based deterministic and neutral processes for the patterns of successional community structure differed among the study sites, we found niche-based deterministic processes are the dominant drivers in structuring plant community assembly regardless of forest age and disturbance in this study. From these results, our study suggests that contemporary forest ecosystems are composed of mosaics of plant communities that are formed by interactions among various processes.

## Introduction

Globally, environmental changes, such as climate change and human impact, are increasingly and simultaneously affecting ecosystem function and biodiversity at local and regional scales (Loreau et al., 2001). Understanding the processes and mechanisms that drive spatio-temporal shifts in community composition is essential in the accurate prediction of future impacts of natural and anthropogenic disturbance (Purschke et al., 2013; Letten et al., 2014) and is a key area of research in the fields of ecology, biogeography, and conservation biology (Chai et al., 2016).

A key challenge in community ecology research is how to disentangle and interpret the role and relative importance of deterministic and neutral processes (Chai et al., 2016; Chun and Lee, 2018). Deterministic hypotheses predict niche-based structuring of local community dynamics by biotic and abiotic filtering, such as by interspecific competition, facilitation, mutualism, and predation, and soil and climate conditions, respectively. Community ecologists and phylogenetic biologists recognize this deterministic process consists of two main drivers such as competition and environmental filtering to structure community assembly. In classical theory, it is believed that interspecific competition suppresses high niche overlap of co-occurring species, while environmental filtering promotes ecological similarity in the areas of high environmental stress (Purschke et al., 2013). Logically, it follows that if ecological niches are phylogenetically conserved, these two opposite processes will produce different phylogenetic structure of communities. That is, competition drives phylogenetic overdispersion (i.e., divergence), whereas environmental filtering leads to phylogenetic clustering (Swenson et al., 2012). Therefore, given this classic framework of successional theory, we can predict community assembly will change from functional and phylogenetic clustering in early succession to functional and phylogenetic divergence at late succession because competition increase and strong environment environmental filtering decrease in relative importance (Connell and Slatyer, 1977). However, recently, Mayfield and Levine (2010) recognized this dichotomous classical framework can't support theories about the relative importance between niche differences and differences in competitive ability, even when ecological niches are phylogenetically conserved. That is, if differences in competitive ability exceed niche differences in communities, competition may exclude the most effective resource competitors. Thus, from this alternative theory, phylogenetic and functional convergence might be predicted rather than divergence, if competition increases through ecological succession (Narwani et al., 2013). However, it is more likely that both processes simultaneously act to affect community structuring, where the relative importance of each in long-term succession depends on the dominant and prevailing environmental conditions (Myers et al., 2013).

On the other hand, the neutral theory of biodiversity suggests that community assemblages are determined by stochastic events, such as dispersal limitation, ecological drift, random disturbance, priority effect, or historical persistence. Although previous studies have reported that niche-based processes is the dominant driver in forest community assemblages (Letcher, 2010; Ding et al., 2012; Purschke et al., 2013; Chai et al., 2016; Chun and Lee, 2018), others have showed that stochastic events may be dominant drivers in ecological succession (Chai et al., 2016).

Ecological succession is the development of community assemblages and is studied to understand the dynamics of ecological community formation and structuring (Purschke et al., 2013) that may facilitate the prediction of responses of ecosystems to environmental change (Chai et al., 2016). Forests are ideal natural experimental systems to assess changes and shifts in community composition during succession (Letcher and Chazdon, 2009). However, many earlier studies on temporal patterns of community assemblage in forest succession tended to focus on species composition and individual traits rather than the integration of multiple traits or phylogenetic relatedness as a proxy of ecological similarity between species (Chai et al., 2016). Species-based approaches used to analyze biodiversity and community structuring in traditional ecology. But the approaches ignore evolutionary and functional adaptive trait response processes in organisms to environment change (Swenson et al., 2012). Recent advances in the complementary areas of phylogenetics and functional trait analysis have provided an improved understanding of patterns of biodiversity and community structure (Kraft and Ackerly, 2010). Thus, previous studies recommended that both diversity need to be considered simultaneously (Purschke et al., 2013; Chai et al., 2016; Chun and Lee, 2018). To date, there have been few studies that comprehensively analyzed the diversity metrics such as species, phylogenetic, and functional diversity with alpha and beta components. Moreover, although it is known that most ecological processes have different effects on forest strata (Ding et al., 2012; Muscarella et al., 2016), few studies have tested changes in community structure among forest strata (i.e., whole, overstory and understory strata) during forest succession.

Studies on the dynamics of community assembly during succession tend to employ chronosequence approaches that assume vegetation patches with different successional stages representing the temporal order of change in structure and composition (Walker et al., 2010; Purschke et al., 2013). Since equivalence of spatio-temporal variations is implicit in chronosequence approaches, they are most appropriate for analysis of linear changes in community structure (Walker et al., 2010). However, community dynamics are not generally linear, as trajectories of spatio-temporal changes and shifts in community dynamics are often mismatched with those changes in soil or environmental conditions (Chai et al., 2016). Due to the lack of suitable long-term monitoring data, most researches for phylogenetic and functional community structure on the effects of succession were limited to static comparisons between both diversity metrics in undisturbed and disturbed vegetation communities (Helmus et al., 2010) or among different successional stages in a chronosequence (Letcher, 2010; Purschke et al., 2013). Most studies documented higher phylogenetic and/or functional dispersion in undisturbed or late successional communities. However, given the known danger and bias of the results from space-for-time substitutions in community ecology researches (Norden et al., 2012), we need to more robustly examine the generality of these patterns with additional actual temporal successional studies (Johnson and Miyanishi, 2008). To date, even few temporal successional studies have concentrated on grass or herbaceous plant communities due to short life cycles of the plant life forms (Purschke et al., 2013; Letten et al., 2014; Conradi et al., 2017). Therefore, it is more needed the ecological researches on the temporal changes of community structure and the drivers in forest ecosystems.

Here, we compared the use of measures of woody plant species diversity with those of phylogenetic and functional diversity in the description of the dynamics and drivers of long-term forest succession in South Korea. Specifically, we (1) quantified species, phylogenetic, and functional alpha and beta diversity for whole, overstory and understory strata at four long-term ecological research sites; (2) tested phylogenetic signals in four functional traits to evaluate the extent to which the phylogenetic relatedness between woody species reflects functional similarity (i.e., niche conservatism); (3) assessed degrees of dispersion in phylogenetic relatedness and functional traits compared with species diversity to understand the roles and relative importance of deterministic and neutral processes in succession; (4) clarified temporal changes in diversity metrics among forest strata and study sites; and finally (5) identified processes that may drive temporal patterns in diversity. Before analyses, we assumed that natural disturbance (i.e., typhoon) acts as an environmental filter and ecological niches are phylogenetically conserved in this study. With these assumptions, we expected that; (1) species diversity has different pattern from phylogenetic and functional diversity and thus species diversity alone cannot reflect spatial and temporal changes in community structure; (2) old growth forest reaching late succession (i.e., Gwangneung) and undisturbed forest (i.e., Mt. Gyebang) would have higher functional and phylogenetic overdispersion; (3) forests (i.e., Mt. Geum and Mt. Halla) damaged by typhoon would have phylogenetic and functional clustering; (4) a forest (i.e., Mt. Geum), which were consecutively disturbed by typhoon, would have more extreme and larger temporal changes in phylogenetic and functional diversity than the other forests; (5) different patterns of phylogenetic and functional diversity would be exhibited among forest strata in all the sites; and finally (6) if measured functional traits in this study are not conserved or have little on community assembly, functional, and phylogenetic diversities would not be highly correlated.

## Materials and Methods

### Study Sites

**Figure 1:**
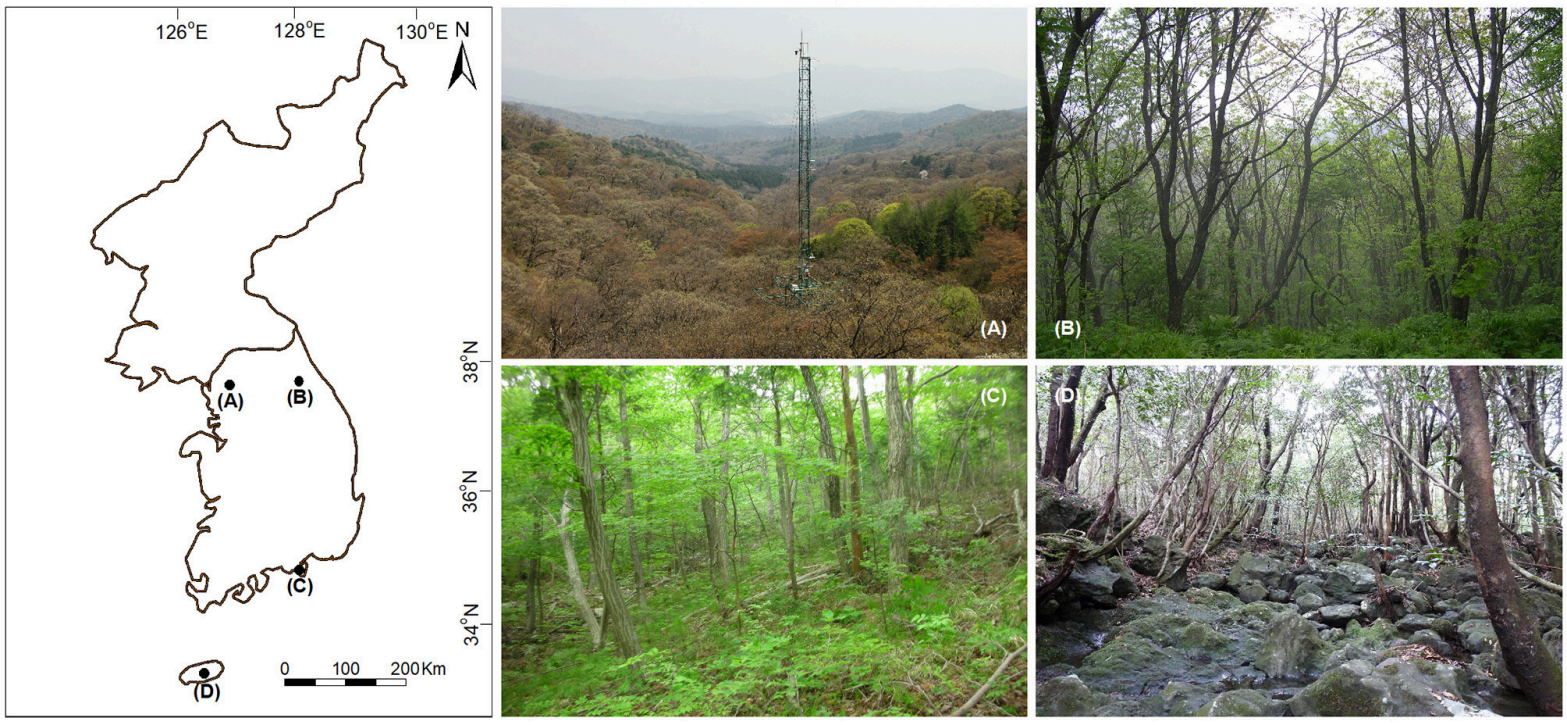
Location and images of the study sites in South Korea. **(A)** Gwangneung, **(B)** Mt. Gyebang, **(C)** Mt. Geum, and **(D)** Mt. Halla.

The study was conducted in four forest long-term ecological research (LTER) sites in South Korea that represented temperature deciduous broadleaved (Gwangneung), temperate coniferous-deciduous mixed (Mt. Gyebang), warm temperate coniferous-deciduous mixed (Mt. Geum), and warm temperate evergreen broadleaved (Mt. Halla) forests in South Korea (Figure 1). The sites were established by the National Institute of Forest Science (NIFoS), which has regularly monitored a range of abiotic and biotic variables, including plant community dynamics, nutrient cycling, biodiversity, and climate change impacts (Lim et al., 2003; Chun et al., 2014). The sites suffered severe damage during the Korean War from 1950 to 1953; however, Gwangneung suffered the least amount of damage and is recognized as old growth forest reaching late successional stage dominated by *Carpinus* and *Quercus* spp. (Lim et al., 2003; Lee et al., 2008). The permanent plots of LTER sites were established in areas not damaged by the Korean War. Mt. Geum in 2003, 2007, and 2014 and Mt. Halla in 2007 were damaged by typhoons (Table 1), where severity of damage differed between the two sites (Chun et al., 2014; Kim et al., 2016).

**Table 1:** Location, climate, soil, and vegetation characteristics at the four forest long-term study sites.

**Characteristic**	**Gwangneung**	**Mt. Gyebang**	**Mt. Geum**	**Mt. Halla**
Latitude	37°44′39″N	37°44′19″N	34°45′35″N	33°18′45″N
Longitude	127°09′22″E	128°27′11″E	127°59′32″E	126°32′31″E
Forest age (years)^a^	>200	70	80	80
Established year	1997	1995	1999	2004
First census year	1998	1997	2000	2005
Damaged year by typhoon^a^	–	–	2003, 2007, 2014	2007
Plot elevation (m)	258	1,100	428	650
Mean annual temperature (°C)	10.7	7.0	13.7	12.7
Mean annual precipitation (mm)	1,397	1,760	1,523	2,648
Forest type	Deciduous broadleaved	Coniferous-deciduous	Coniferous-deciduous	Evergreen broadleaved
Dominant tree species	*Carpinus laxiflora* *Cornus controversa* *Quercus serrata*	*Pinus densiflora* *Acer psedosieboldianum* *Quercus mongolica*	*Chamaecyparis obtusa* *Carpinus tschonoskii* *Quercus serrata*	*Camellia japonica* *Carpinus laxiflora* *Quercus acuta*
Total woody species^b^	36 (32–34)	35 (32–35)	43 (32–42)	48 (35–47)
Stem density (ha^−1^)^c^	1,513–1,857	2,542–2,988	2,582–3,743	4,832–6,144
Parent rock	Granite gneiss	Granite gneiss	Granite	Trachybasalt
Soil texture	Silt loam, loam	Silt loam, loam	Silt loam, loam	Silt loam
pH (at 0–10 cm)	4.91	5.44	4.52	5.36

a *Forest age and damaged year by typhoon for each site was cited from Cho et al. (2007), Lee et al. (2008), Chun et al. (2014), and Kim et al. (2016) and the “damaged year by typhoon” means the year in which typhoons occurred during census periods*.

b *Total woody species is the number of all woody species recorded in the censuses, and numbers in parentheses are minimum and maximum values*.

c *Stem density is the minimum and maximum stem density recorded in the censuses*.

Woody plants at Gwangneug, Mt. Gyebang, and Mt. Geum were surveyed over 15 years and over 10 years at Mt. Halla. A census of woody plant species was collected at each study site every 5 years in a 1-ha (100 m × 100 m) permanent plot. The permanent plot was divided and marked into 100 quadrats measuring 10 × 10 m^2^. All standing woody stems ≥2 cm diameter at breast height (DBH) were identified to species, tagged, mapped, and measured for DBH and height following a standardized protocol (Lim, 1998; Chun et al., 2014). We converted the 10 × 10 m^2^ quadrat data to 25 quadrat data of 20 × 20 m^2^ at each site. And in order to explore the influence of forest strata, we also divided all stems into overstory and understory strata. Overstory and understory strata included all tree stems with DBH ≥ 10 cm and 2 cm ≤ DBH < 10 cm, respectively. In this study, we analyzed community structure and compositional turnover only with 20 x 20 m^2^ quadrat data because we cannot obtain the entire and intact phylogenetic and functional diversity values for both overstory and understory strata with 10 × 10 m^2^ quadrat data.

### Functional Traits

Four functional traits associated with fundamental functional trade-offs under successional or environmental change pressure (Garnier et al., 2016) were selected and comprised maximum height (m), leaf size (cm), seed mass (mg), and wood density (g/cm^3^) and obtained for the recorded woody plant species from published literature and open databases (Supplementary Data A1). In general, maximum height is positively correlated with competitive dominance in limited light conditions and time to reproduction (Muscarella et al., 2016). Leaf size is related to resource acquisition and energy fluxes (Edwards et al., 2014). In this study, we used the sum of leaf length and width as a proxy of leaf area because previous study in our sites reported that leaf area was well correlated with the sum of leaf length and width (Lee et al., 2008). Seed mass is associated with a trade-off between seed size and reproductive ability (Moles and Westoby, 2006), and smaller seeds have the advantages of dispersal and greater longer-term survival in the soil (Muscarella et al., 2016). Wood density represents a trade-off between structural investment and growth and mortality rates (Swenson et al., 2012). High wood density implies high structural investment of a tree species and low growth and high survival rates, whereas low wood density indicates low structural investment and high growth and mortality rates (Swenson et al., 2012). A mean value of a trait for a given woody species was used and the mean trait values were assigned for functional diversity analysis. Thus, the present study does not reflect trait variation among and within woody species (Swenson et al., 2012).

### Phylogenetic and Functional Tree Construction

A community phylogeny of woody plant species at each study site was derived from an expanded and updated version of a vascular plant phylogeny generated by Zanne et al. (2014), PhytoPhylo megaphylogeny (Qian and Jin, 2016) and any species that were absent from PhytoPhylo were assigned using the S. PhyloMaker function with Scenario 3 in R version 3.4.3 with the *phytools* package.

In addition to constructing phylogenetic trees for each of the four study sites, we also constructed a phylogenetic tree including all of the woody plant species in four LTER sites to measure phylogenetic signals. Dendrogram for the four functional traits of the communities in each of the study sites was constructed using UPGMA hierarchical clustering, based on a Euclidean distance matrix.

We used five phylogenetic trees and four functional trait dendrograms to quantify the changes in phylogenetic and functional diversities during forest succession in the four study sites (Supplementary Figure A1).

### Phylogenetic Signal in Functional Traits

We assessed the degree of similarity of phylogenetic relatedness with the four functional traits using Pagel's λ (Pagel, 1999) and Blomberg's *K* (Blomberg et al., 2003). If Pagel's λ = 0, phylogenetic dispersion is different from that of functional traits and λ = 1 indicates that the trait distribution follows Brownian motion. Moreover, Blomberg's *K* > 1 or *K* < 1 represents that the phylogenetic signal in a trait was higher or lower, respectively, than expected under Brownian motion. The significance of *K* and λ was tested by randomly arraying the trait data on community phylogeny 1,000 times to produce a null distribution.

### Diversity Metrics

Alpha species diversity was calculated as species richness (number of species) in each quadrat. Beta species diversity was calculated by two methods such as turnover between-quadrats in a same census (i.e., spatial beta diversity) and between-censuses in a same quadrat (i.e., temporal beta diversity) using Bray-Curtis dissimilarity index. Phylogenetic and functional alpha community structure in a quadrat of each census was calculated using abundance-weighted net relatedness index (NRI) (Webb et al., 2008) as follows:

$$NRI=\frac{-(MPD_{obs}-meanMPD_{null})}{sdMPD_{null}}$$

where *MPD* is mean pairwise phylogenetic or functional distance between co-occurring species in each quadrat; and, *MPD*_*obs*_, mean *MPD*_*null*_, and sd *MPD*_*null*_ are observed value, mean value and standard deviation based on a null model, respectively. The null model randomly shuffled species names across the tips of the phylogenetic trees or functional trait dendrograms 1,000 times. This process randomizes the phylogenetic or functional relatedness of species to one another while maintaining the observed community data matrix. Thus, this null model retains the observed species richness, species occupancy rate, and abundance in each randomization (Swenson et al., 2012).

To examine phylogenetic and functional beta community structure between paired quadrats of each census at each study site, we calculated the abundance-weighted pairwise phylogenetic and functional dissimilarity matrix (*D*_*pw*_) and the standardized effect size (*S.E.S*.) of *D*_*pw*_ (Swenson et al., 2012) as follows:

$$D_{pw}=\frac{1}{2}\left(\sum_{i=1}^{S_{a}}f_{i}\bar{\delta_{ib}}+\sum_{j=1}^{S_{b}}f_{j}\bar{\delta_{ja}}\right)$$

where *f*_*i*_ and *f*_*j*_ are the relative abundance of species *i* and *j*, respectively; and $\bar{\delta_{ib}}$ and $\bar{\delta_{ja}}$ are the mean pairwise phylogenetic or functional distances between all species in the same quadrat at times *b* and *a*, respectively, and species *i* and *j*, respectively, in a quadrat at times *a* and *b*, respectively.

$$S.E.S.D_{pw}=\frac{-(D_{pw\;obs}-meanD_{pw\;null})}{sdD_{pw\;null}}$$

where *D*_*pw obs*_, *mean D*_*pw null*_, and *sd D*_*pw null*_ are the observed value, mean value and standard deviation based on a null model, respectively. The phylogenetic and functional *D*_*pw*_ and *S.E.S.D*_*pw*_ between paired censuses in a quadrat were also calculated using the method above. The null model was generated in the same way as NRI.

The COMSTRUCT and COMDIST functions in Phylocom 4.2 (Webb et al., 2008) were used to generate null models and calculate *NRI* and *S.E.S*. *D*_*pw*_, respectively, using the phylogenetic trees and functional trait dendrograms constructed in section “Phylogenetic and Functional Tree Construction.”

Moreover, community-weighted mean trait values for each trait in each quadrat at each census based on relative abundance and average trait values of species were calculated to evaluate the shift in a single trait during forest succession. All the diversity metrics were calculated for each of forest strata such as whole, overstory and understory strata.

Linear mixed effect modeling was used to evaluate the differences in mean values of species, phylogenetic and functional diversity and community-weighted mean trait values among forest successional stages by including plot as a random factor at each site using R package *lme4* and Tukey's *post-hoc* multiple comparison test was performed using *multcomp* package in R version 3.4.3. We used Kernel density estimation to examine the degree of temporal shift in species, phylogenetic, and functional composition during forest succession using SigmaPlot version 14.0.

## Results

A total of 99 woody plant species belonging to 37 families and 59 genera were recorded from the four forest study sites (Supplementary Data A1). The highest number of species occurred at the Mt. Halla site where there were 44 species representing 33 genera from 26 families, and the lowest number of species was recorded from the Mt. Gyebang site (35 species representing 26 genera from 20 families).

### Phylogenetic Signal in Functional Traits

We found that values of Pagel's λ were greater than Bloomberg's *K* and all values were <1 for the four functional traits (Table 2). However, all the traits exhibited significant phylogenetic signals. These results represent that using the phylogenetic dispersion as a rough substitute of functional dispersion is proper for woody plant species in this study.

**Table 2:** Phylogenetic signal in functional traits using Pagel's λ and Blomberg's *K* statistic.

**Functional trait**	**Pagel's λ**	**Blomberg's *K***
Maximum height (m)	0.660^**^	0.115^*^
Leaf size (cm)	0.837^***^	0.086
Seed mass (mg)	0.999^***^	0.259^**^
Wood density (g/cm^3^)	0.982^***^	0.419^***^

* *P < 0.05*,

** *P < 0.01*,

*** *P < 0.001*.

### Changes of Diversity Metrics During Censuses

**Figure 2:**
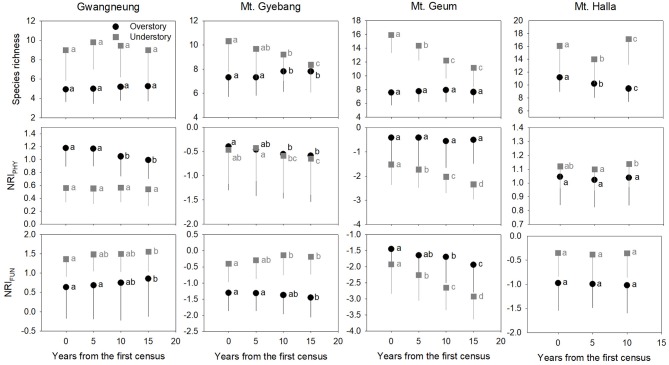
Temporal changes in species, phylogenetic, and functional alpha diversity at overstory and understory strata. Letters represent significant differences (*P* < 0.05) between censuses. Mean values of each metric of diversity in each census of a study site significantly differed from zero using a one-sample *t*-test, indicating alpha diversity of the three diversity metrics was not random. Black and gray lines indicate standard deviations. NRI_PHY_ and NRI_FUN_ represent phylogenetic and functional alpha diversity, respectively.

**Figure 3:**
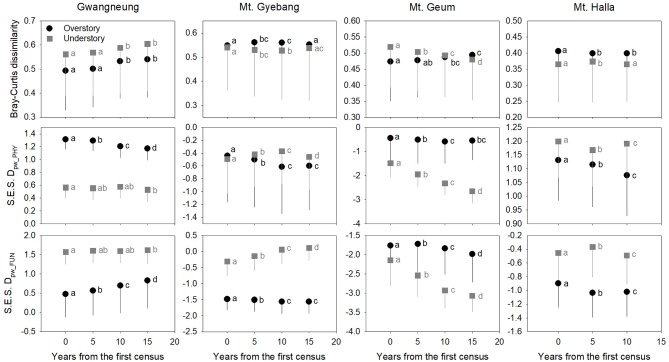
Temporal changes in species, phylogenetic, and functional beta diversity between paired quadrats at overstory and understory strata. Letters represent significant differences (*P* < 0.05) between censuses. Mean values of each diversity metric in each census of a study site significantly differed from zero using a one-sample *t*-test, indicating beta diversity (turnover) of the three diversity metrics was not random. Black and gray lines indicate standard deviations. S.E.S. D_pw_PHY_ and S.E.S. D_pw_FUN_ represent phylogenetic and functional beta diversity, respectively.

Although there were some exceptions, the temporal patterns of alpha and beta components of species, phylogenetic and functional diversity were generally different, whereas phylogenetic and functional diversity had similar patterns between overstory and understory strata at all study sites (Figures 2, 3). At Gwangneung, species richness were not different during censuses, whereas Bray-Curtis dissimilarity increased during censuses at overstory and understory strata. In Mt. Gyebang, species richness increased and decreased at overstory and understory strata, respectively, whereas Bray-Curtis dissimilarity was not changed through time. Phylogenetic alpha and beta diversity decreased at overstory strata and functional alpha and beta diversity increased at both strata in Gwangneung and Mt. Gyebang. Alpha and beta components of three diversity metrics simultaneously decreased at understory strata in Mt. Geum. Alpha and beta components of three diversity metrics were mostly decreased in the second census and then increased in the third census in Mt. Halla despite of some exceptions.

Contrary to our hypotheses, at Gwangneung, phylogenetic and functional alpha and beta diversity showed clustering patterns with close lineages and lower turnover between close lineages than expected. Mt. Geum also exhibited different results from our prediction. The overdispersion pattern and higher turnover in phylogenetic and functional diversity were shown and the tendency was more conspicuous at the understory strata in Mt. Geum. Moreover, at Mt. Halla, the both diversity metrics during censuses exhibited different patterns such as phylogenetic clustering and functional dispersion in contrast to the other sites. However, at Mt. Gyebang, both diversities were overdispersed with distant lineages and higher turnover between distant clades than expected as our expectation.

**Figure 4:**
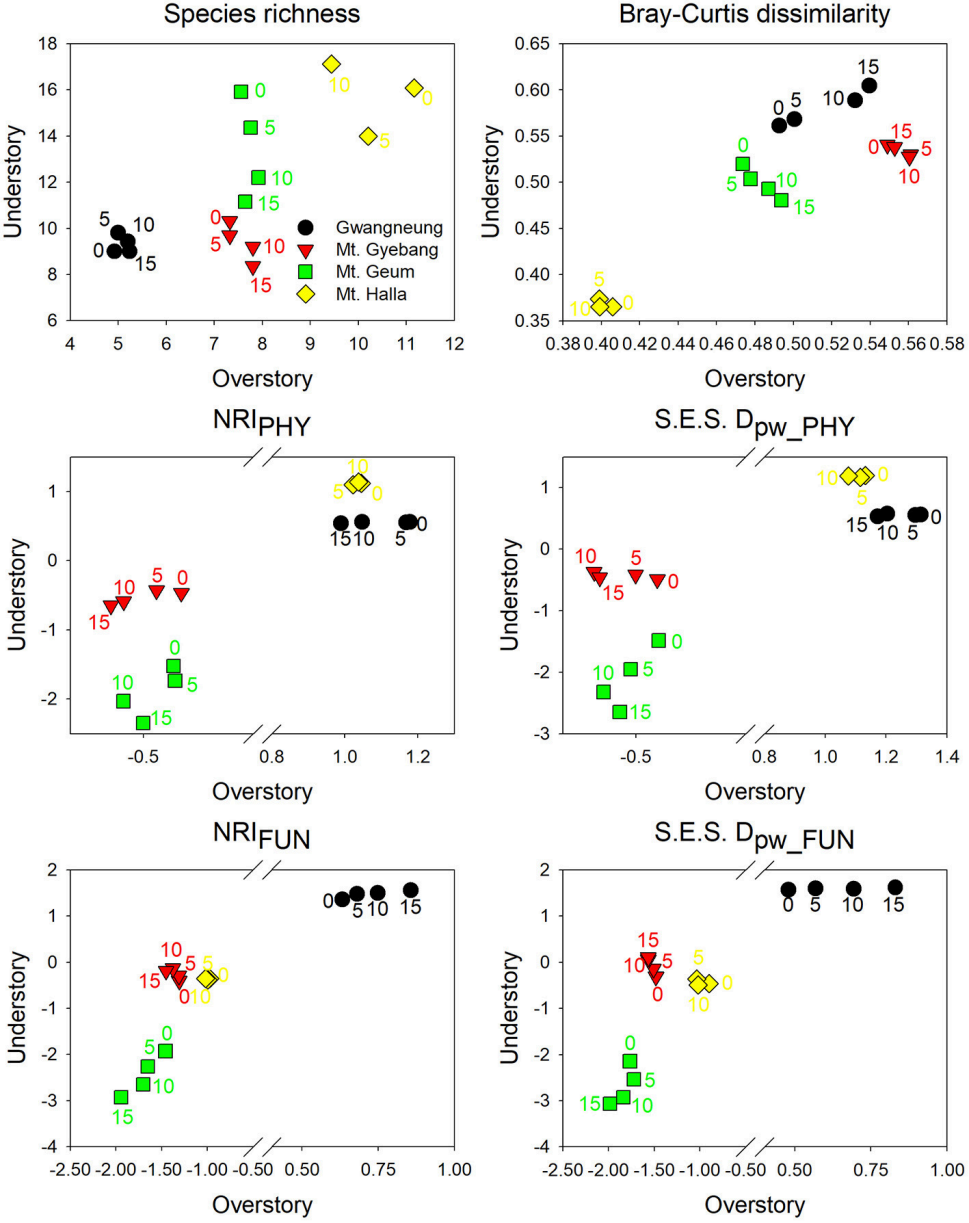
Temporal changes in alpha and beta components of species, phylogenetic, and functional diversity at overstory and understory strata. NRI_PHY_, NRI_FUN_, S.E.S. D_pw_PHY_, and S.E.S. D_pw_FUN_ represent phylogenetic and functional alpha diversity and phylogenetic and functional beta diversity, respectively. The numbers indicate years elapsed from the first census.

The temporal patterns between alpha and beta components in species diversity were different from the patterns of the both components in phylogenetic and functional diversity during censuses at all the sites. However, the patterns between the both components in phylogenetic and functional diversity were similar across the censuses at all the sites except for Mt. Halla (Figure 4).

The temporal patterns of whole strata for alpha and beta components of three diversity metrics were similar with those of understory strata at Mt. Gebang, Mt. Geum and Mt. Halla, whereas the patterns of both components in three diversity metrics for whole strata were similar with those of overstory strata at Gwangneung (Supplementary Figures A2, A3).

### Community-Weighted Mean Traits

**Figure 5:**
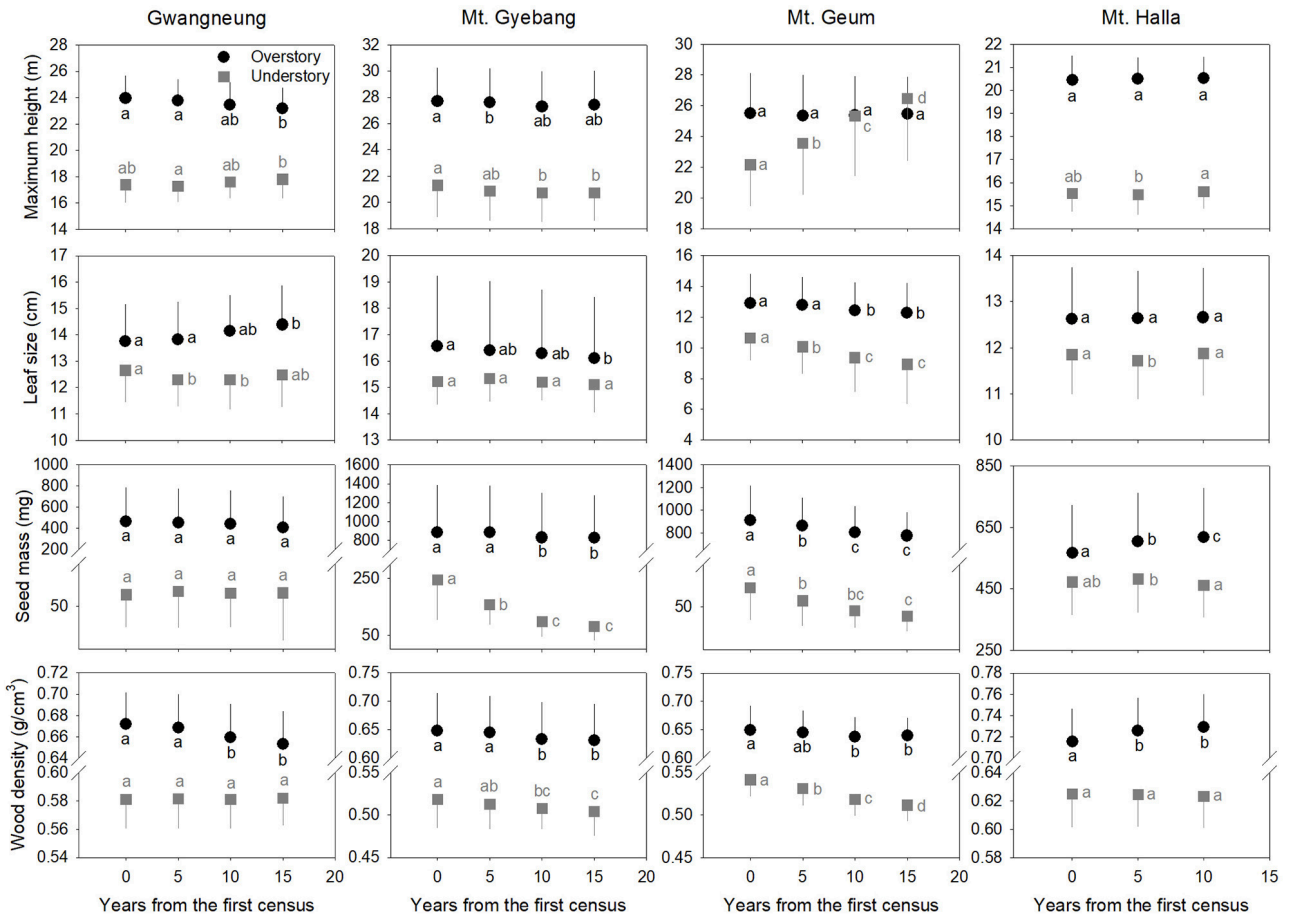
Community-level mean values for the four functional traits through time at overstory and understory strata. Letters represent significant differences (*P* < 0.05) between censuses. Black and gray lines indicate standard deviations.

Community-weighted mean trait values were different between overstory and understory strata at all the sites (Figure 5). At Gwangneung, mean trait values of maximum height had different patterns between overstory and understory strata over time. And leaf size and wood density increased and decreased, respectively, at overstory vegetation. At Mt. Gyebang, mean trait values of seed mass and wood density decreased at both overstory and understory strata. In Mt. Geum, mean trait values of maximum height increased at understory vegetation over time, whereas leaf size, seed mass and wood density decreased at both overstory and understory strata through time. Mean trait values of all the functional traits except for seed mass at overstory strata were not changed over time at Mt. Halla. The temporal patterns of community-weighted mean trait values for whole strata were different from those of overstory and understory strata at all the sites despite of some exceptions (Supplementary Figure A4).

### Temporal Turnover of Diversity Metrics Between Censuses

**Figure 6:**
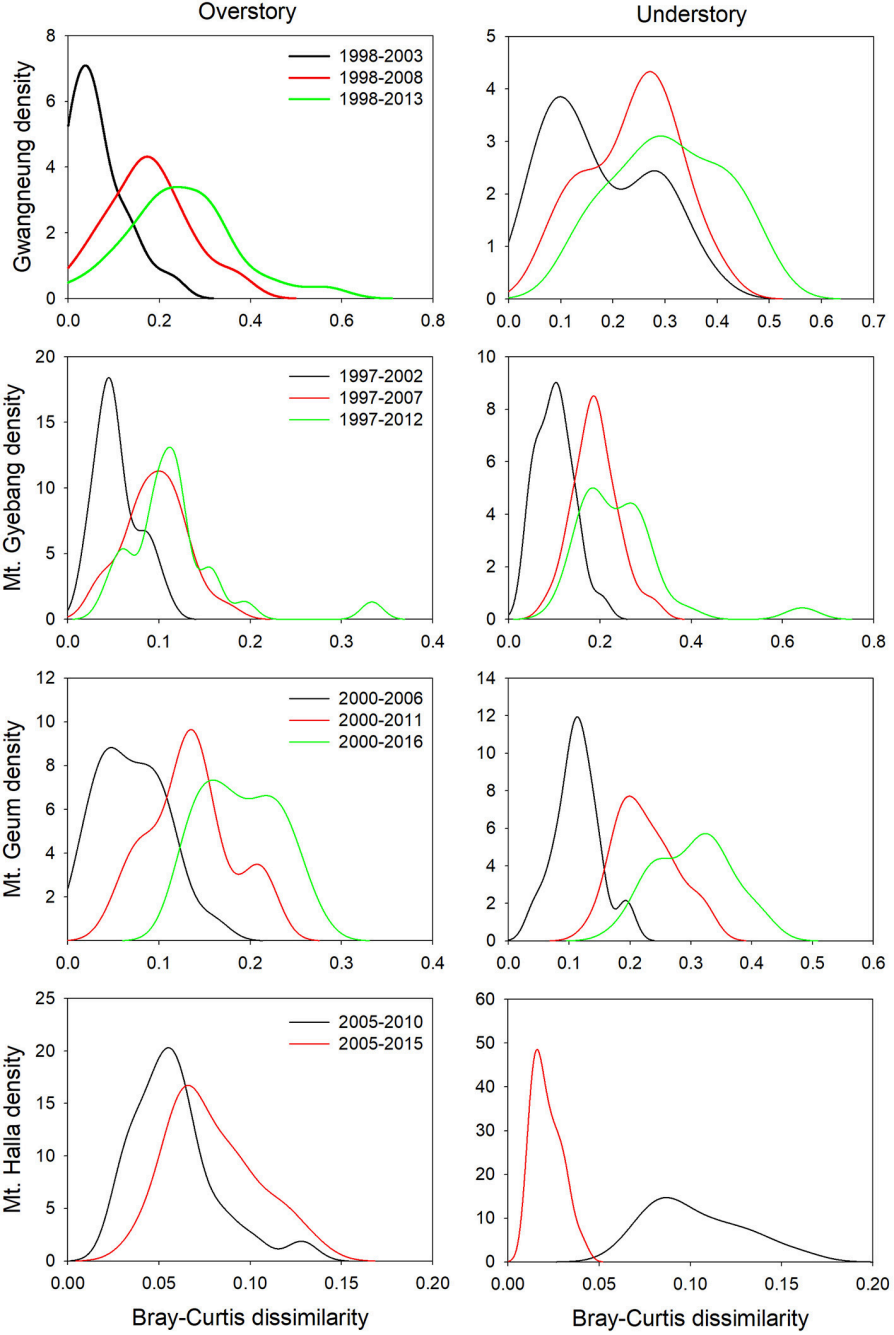
Temporal turnover of species composition between paired censuses in a quadrat quantified by Bray-Curtis dissimilarity. The plots were drawn by Kernel density estimation.

**Figure 7:**
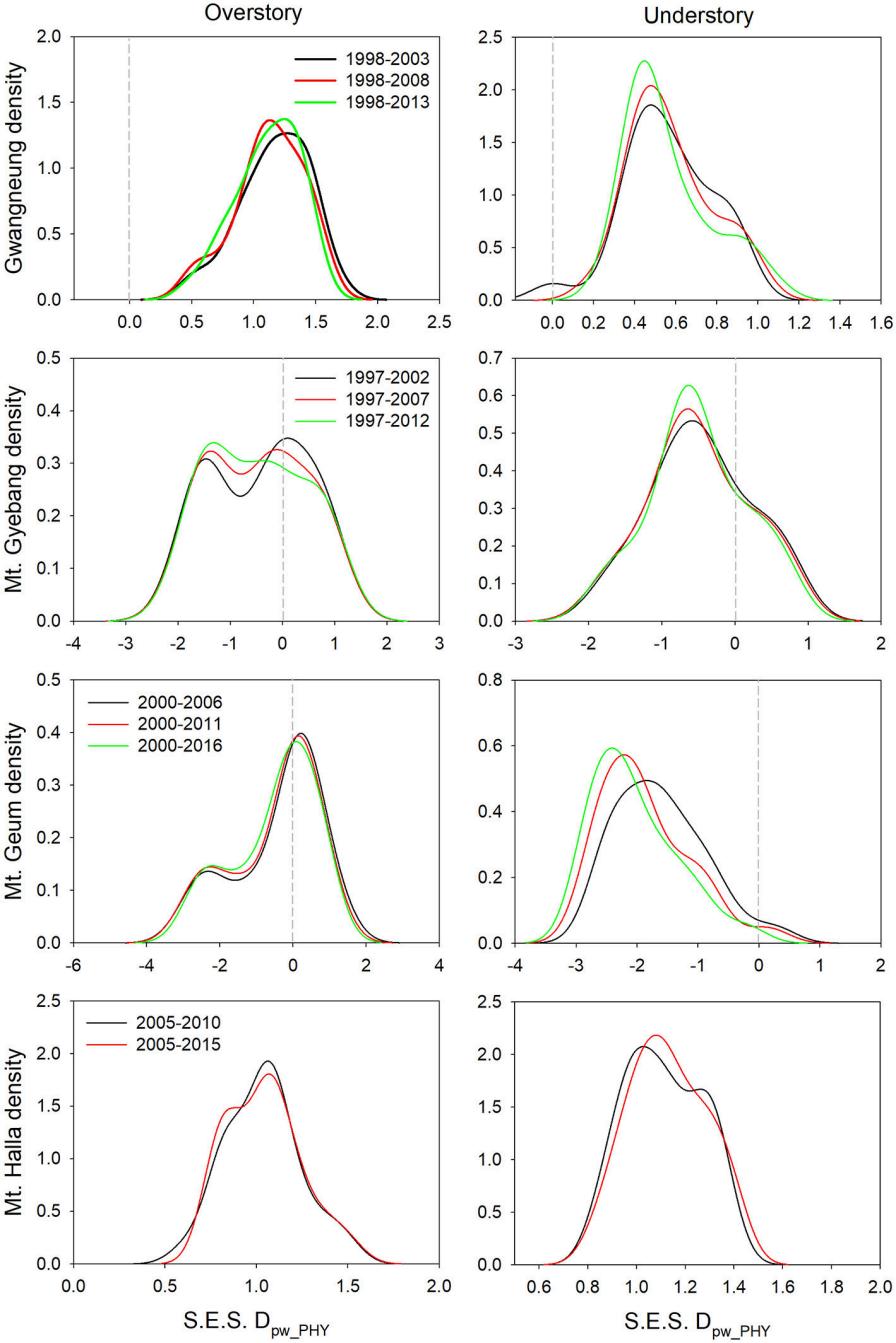
Temporal turnover of phylogenetic composition between paired censuses in a quadrat quantified by S.E.S. D_pw_PHY_. The plots were drawn by Kernel density estimation.

**Figure 8:**
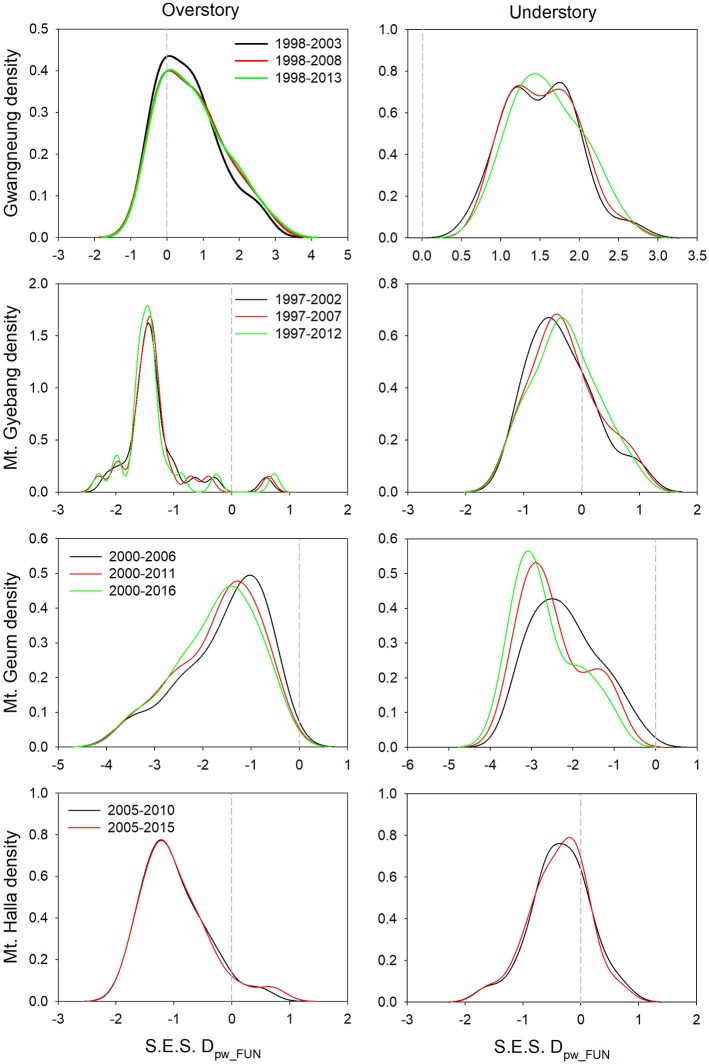
Temporal turnover of functional composition between paired censuses in a quadrat quantified by S.E.S. D_pw_PHY_. The plots were drawn by Kernel density estimation.

Although there were substantial temporal declines in species composition at all sites (Figure 6), within-quadrat temporal turnover of phylogenetic and functional diversity between paired censuses differed among the sites and between forest strata (Figures 7, 8). There was little phylogenetic and functional turnover between censuses at Gwangneung, Mt. Gyebang, and Mt. Halla. However, it was greater at Mt. Geum, especially for understory vegetation. We found that phylogenetic turnover at Gwangneung and Mt. Halla was less than expected, whereas it was greater than expected at Mt. Gybang and Mt. Geum. We also found higher than expected functional turnover at all sites except Gwangneung. Trends of phylogenetic and function turnover differed at Mt. Halla. The within-quadrat temporal turnover of three diversity metrics for whole strata between censuses were also different from those of overstory and understory strata at all the sites despite of some exceptions (Supplementary Figure A5).

## Discussion

Using empirical data, we examined the long-term succession of phylogenetics and functional traits with three diversity metrics in four forests, in South Korea. And this study also contributes to previous field-based temporal successional studies (Swenson et al., 2012; Muscarella et al., 2016) in contrast to time-for-space approaches suggested in previous many studies (e.g., Purschke et al., 2013; Chai et al., 2016) that assume drivers of spatial gradients of diversity also drive temporal changes. Our study of species, phylogenetic, and functional trait diversity showed variable successional patterns among forest strata and the different temperate forests. We also found that the changes in species alpha and beta diversity differed from those of phylogenetic and functional trait diversity at each census time point at three study sites (Gwangneung, Mt. Gyebang, and Mt. Halla). At Mt. Geum, however, there were similar successional changes among the three diversity metrics except for the functional beta diversity at overstory vegetation. We found there were little or no temporal changes in phylogenetic and functional turnover between paired censuses in quadrats at all sites except Mt. Geum, but there were increasing dissimilarities in species composition with time at all study sites.

### Phylogenetics as Surrogate Measure of Functional Traits

In the analysis of phylogenetic signals of the four functional traits, we found that values for Pagel's λ and Blomberg's *K* were <1 for all traits and were significant. These results indicate that functional traits were more labile than expected and that there was approximate matching between phylogenetic and functional diversity (Swenson et al., 2012). Previous studies also reported similar results with not-perfect (or rough) alignment but significant relationships between phylogenetic and functional diversity in other forest ecosystems (Kraft and Ackerly, 2010; Yang et al., 2014; Chun and Lee, 2018). Moreover, our study supports the idea that phylogenetic diversity may be used a crude surrogate measure of functional diversity if low but significant phylogenetic signals in traits exist. The fact that stronger environmental filtering than competitive exclusion leads to phylogenetic clustering is possible when the premise of significant phylogenetic signal in the traits involved in assembly processes is fulfilled (Mayfield and Levine, 2010). Therefore, many community ecologists recognize the necessity of the test of phylogenetic signals in functional traits. And they also suggest using both diversity metrics simultaneously to complement each other (Swenson et al., 2012).

### Successional Changes in Metrics of Diversity

At Gwangneung, there were no temporal changes of species alpha diversity at all forest strata, whereas species turnover increased with time. However, phylogenetic and functional alpha diversity were not changed during censuses at whole strata, whereas the patterns were different between overstory and understory strata. Overstory vegetation decreased phylogenetical clustering but was functionally more clustered, whereas understory vegetation was phylogenetically stable but functionally more clustered through time. For measures of beta diversity, we found that species turnover increased with time, phylogenetic turnover decreased, and functional turnover increased at all forest strata. We found that community-weighted mean values of the four functional traits did not change with time at understory vegetation, whereas mean trait values of seed mass was not changed and the other trait values were changed with time at overstory vegetation. These results suggest that the structure of the phylogenetic and functional communities of the forest at Gwangneung were stable at whole community, due to lack of disturbance, as indicated by high levels of phylogenetic and functional clustering and lower phylogenetic and functional turnovers (Gerhold et al., 2015; Chun and Lee, 2018). Indeed, previous studies have described the forest at Gwangneung as an old growth and late successional community (Lim et al., 2003; Chun et al., 2014). We suggest that the high phylogenetic and functional relatedness of the woody species communities at the study site through time may be a result of a deterministic process of environmental filtering made in a very old past. However, our results also highlight that the responses of phylogenetic and functional diversity through time can differ between overstory and understory strata, although the whole strata including overstory and understory strata are phylogenetically and functionally static. Recent studies also reported that phylogenetic and functional structures of communities also change with forest strata but with different tendencies (Letcher, 2010; Ding et al., 2012).

Species richness decreased at whole and understory strata but increased at overstory vegetation during censuses at Mt. Gyebang. And phylogenetic alpha diversity decreased at all forest strata but functional alpha diversity differed among forest strata. These results indicate that species richness had no impact on phylogenetic and functional alpha diversity (Figure 2). Species turnover decreased during censuses at whole strata but species turnover was not different between the first and last censuses for overstory and understory strata. Phylogenetic and functional turnovers decreased at overstory vegetation, whereas the both turnovers increased at whole and understory strata. These results show a slow but continual divergence and convergence of functional traits at overstory and understory communities, respectively, during forest succession (Muscarella et al., 2016). And phylogenetic divergence was shown during censuses at all forest strata with turnover between phylogenetically distant lineages. We found that mean trait values for seed mass and wood density decreased at whole strata over time in Mt. Gyebang, however mean values of all traits decreased at both overstory and understory strata except for two cases (i.e., maximum height and leaf size at overstory and understory strata, respectively). These results indicate that species with smaller seeds and leaf size at overstory vegetation were better adapted to the nutrient-poor and drier conditions that develop during succession (Purschke et al., 2013). However, our results at Mt. Gyebang conflict with previous studies that have showed small-seeded plants with low wood density flourish in communities of early successional stages and plant species with large seeds dominate with high wood density in later successional stages, according to the competition-colonization trade-off theory (Purschke et al., 2013). However, indeed, the abundance ratio of species with large seeds, such as *Quercus mongolica* and *Coryllus heterophylla*, decreased with time, whereas abundance of small-seeded species, such as *Acer pseudosieboldianum*, increased at Mt. Gyebang (Supplementary Data A1), possibly due to interactions between species with these functional traits and the local environment, as has been shown in other studies (e.g., Ma et al., 2013). Simultaneously, the abundance ratio of species with large seed, small leaf and low density such as *Pinus densiflora, P*. *koraiensis*, and *Abies holophylla* increased at both overstory and understory strata by recruitment and thus leaf size, seed mass and wood density decreased at overstory and understory strata during censuses at Mt. Gyebang.

In contrast to other study sites, the patterns of the three diversity metrics at whole strata were similar over time in Mt. Geum. The alpha and beta components of three diversity metrics decreased through time at whole strata (Figures 2, 3). These results represent that overdispersion increased in phylogenetic and functional alpha community structures over time and functional divergence between distant lineages are higher and faster than expected. And the results from phylogenetic and functional turnover between first and subsequent censuses reinforce these phylogenetic and functional divergences at Mt. Geum (Supplementary Figures A4, A5). And these patterns were stronger at understory community than at overstory community in Mt. Geum (Figures 7, 8). These results indicate that the continuous disturbance by typhoon removed more understory stems than overstory stems (Supplementary Data A1). We found that community-weighted mean values of maximum height increased at whole and understory strata, whereas leaf size and wood density decreased at all forest strata. And maximum height and seed mass at overstory and whole strata, respectively, were not changed with time. Similar effects of disturbance during succession on maximum height (especially, at understory strata), seed mass and wood density showed across various ecosystems in northwest Europe have been reported by Douma et al. (2012). Light competition leads to increases in maximum height at understory strata and shifts to fast growing woody species with low wood density at all forest strata (Douma et al., 2012). It is also possible that the frequent disturbance by typhoons in 2002, 2003, 2007, and 2014 at Mt. Geum, which caused an increase in the mortality rate of broadleaved species (e.g., *Stewartia pseudocamellia, Styrax japonicas*, and *Quercus serrata*) and an increase in recruitment rate of conifer species such as *Chamaecyparis obtusa* may have contributed to the reduction of leaf size, seed mass and wood density over time (Chun et al., 2014; Kim et al., 2016).

At Mt. Halla, we found species and phylogenetic alpha diversity decreased between the first and second censuses and increased between the second and third censuses at whole and understory strata, whereas there was no change in phylogenetic alpha diversity at overstory vegetation and functional alpha diversity at all forest strata, and turnover of species composition was not changed while turnover of phylogenetic relatedness and functional traits decreased when we compared the last census with the first census. These results indicate phylogenetic relatedness may not correlate with functional trait diversity and there may have different assembly processes between phylogenetic and functional diversity (Swenson et al., 2012; Muscarella et al., 2016). When we compared the last census with the first census, community-weighted mean values of all the traits were not changed at whole strata. Mean values of seed mass and wood density increased only at overstory vegetation (but not changed at understory vegetation), whereas the other traits were not changed at overstory and understory strata. In general, early colonization is related to high dispersal ability and fecundity, whereas species with larger seeds, which have higher establishment ability and tolerance of impoverished resource conditions, occur at later successional stages (Mason et al., 2012). Therefore, our results from overstory community in Mt. Halla highlight the role and importance of seed size and wood density in competition-colonization trade-off and successional niche theories. And also these changes in seed size and wood density may be related to the occurrence of typhoons between the first and second censuses in this site that may have eliminated species with low population size, smaller seed and lower wood density (Chun et al., 2014).

### Community Dynamics

Traditional theory of community dynamics proposes that abiotic processes dominate in early successional stages, whereas biotic processes become more important at the later stages (Muscarella et al., 2016). Our results showed that temporal changes differed among the three diversity metrics, between the alpha and beta scales of diversity, among the three forest strata and among the four study sites, indicating that the metrics and scales of estimating diversity and forest strata were complementary since they each provided additional information (Ding et al., 2012; Purschke et al., 2013; Muscarella et al., 2016; Chun and Lee, 2018). Despite these differences, our results indicate that the key structuring process in woody plant assemblages at the sites may be niche-based deterministic, such as environmental filtering and competitive exclusion, because phylogenetic and functional alpha and beta diversity deviated from zero value (Figures 2, 3). Our findings are consistent with recent studies that report the dominance of niche-based deterministic processes in forest succession (Letcher, 2010; Ding et al., 2012; Purschke et al., 2013; Chai et al., 2016).

Contrary to our expectation, at Gwangneung, there was functional and phylogenetic convergence during forest succession possibly as a result of selection pressure from environmental conditions. Indeed, gaps formed by the death of large and old-growth trees create contrasting light environments of open and closed microhabitat and thus similar types of environments are repeatedly formed (Lim et al., 2003). It is likely that these two repeated microhabitats drive the communities in each toward compositions that are phylogenetically and functionally more closely related (Lim et al., 2003; Chun et al., 2014). In general, harsh environments lead to phylogenetic and functional clustering (Letten et al., 2014), and at Gwangneung, rainfall is concentrated between July and August and water availability is limited since soil water is rapidly depleted after the rainy period (Hwang et al., 2008). Therefore, water may be a key limiting factor for plant growth at Gwangneung (Hwang et al., 2008; Laiju et al., 2012) and impacts on colonizing woody plants may be greater due to extended periods of dry soil conditions (Hwang et al., 2008; Laiju et al., 2012). Indeed, several dominant woody plants, such as *Quercus serrata* and *Styrax japonica*, have well-developed root systems with large amounts of fine roots and a deep main root to facilitate rapid water uptake following rainfall events (Laiju et al., 2012).

Although phylogenetic and functional divergences were recorded at Mt. Gyebang as our expectation and Mt. Geum contrary to our hypothesis, drivers of ecological processes appeared to differ. The relative importance of competitive exclusion may become greater than environmental filtering at Mt. Gyebang due to the high elevation (Table 1) that may then lead to phylogenetic and functional overdispersion as a result of removal of related, ecologically similar species (Mayfield and Levine, 2010). Chun and Lee (2018) inferred that phylogenetic and functional overdispersion patterns of plant species at a site close to Mt. Gyebang tended to be mainly induced by competitive exclusion at higher elevations, and it has been shown that high environmental heterogeneity at small spatial scales, such as with quadrats, induces functionally and phylogenetically dissimilar species assemblages (Yang et al., 2014).

In contrast, at Mt. Geum, the accelerated phylogenetic and functional divergence seemed to be induced by disturbance from frequent natural events. Damage caused by multiple typhoons at Mt. Geum probably removed phylogenetically and functionally similar species and triggered the development of highly dissimilar communities over phylogenetic and functional trait space. Competitive exclusion of functional groups also accelerates overdispersion (Mayfield and Levine, 2010) and may have been triggered at Mt. Geum by typhoons that promoted abundance of species with traits tolerant of the impacts of typhoon events. Indeed, the 10 species that disappeared during the censuses (Table 1) shaped the community composition (Supplementary Data A1). Our data show that the composition of the woody plant communities at Mt. Geum were a result of niche-based deterministic processes and stochastic events and support previous research that showed natural disturbances are the primary influence on populations of forest species and drive higher and more rapid functional turnover with time (Swenson et al., 2012).

Contrary to our expectation, phylogenetic convergence and functional divergence were recorded at Mt. Halla, where soils were interspersed with rocky outcrops that constitute two types of microhabitat (Chun et al., 2014). *Quercus acuta, Carpinus laxiflora*, and *Distylium racemosum* that belong to the Hamamelidae, and *Camellia japonica, Cleyera japonica*, and *Eurya japonica* that belong to the Theales, are phylogenetically close. These woody species inhabit both types of microhabitat (Chun et al., 2014) present at Mt. Halla and were the most abundant in the site (Supplementary Data A1). However, there are extreme differences in their seed mass despite the phylogenetic closeness (Supplementary Data A1, Supplementary Figure A1). Therefore, these differences likely led to functional divergence at Mt. Halla. It is also likely that a typhoon removed highly dispersed functional groups with low abundance. Indeed, eight distantly related species of low abundance disappeared during the censuses at Mt. Halla (Supplementary Data A1). Our results indicate that niche-based deterministic processes, such as habitat conditions, and stochastic events, such as natural disturbance, contributed to the formation of the woody plant community assemblage at Mt. Halla.

Our study of phylogenetic and functional diversity of forest woody plant species emphasized the complex and unpredictable interactions between community assemblage and structuring processes (Mayfield and Levine, 2010) and revealed the roles of the different processes in forest community ecology. The patterns of successional community composition may be explained by niche-based deterministic and neutral processes, and the role and relative importance of these two processes differed among the study sites (Chase and Myers, 2011). We tested for differences in temporal patterns of diversity at forest strata and found temporal changes were different although community structures were similar among the strata in the sites through time (e.g., phylogenetic and functional clustering and lower turnover in Gwangneung). Therefore, our study suggests that forest strata need to be considered when we study community structures of plant assemblages in forest ecosystems. Moreover, we hypothesized that Mt. Geum, which is consecutively disturbed by typhoon, would have more extreme and larger temporal changes in phylogenetic and functional diversity than the other forests. And we found that the temporal changes of the both diversity for understory strata at Mt. Geum were more extreme and larger than those of understory strata in the other sites but there were no more extreme and larger differences among overstory strata in the study areas. Recent researches have reported that community structures of plants may differ with forest strata and such a difference can be accelerated by disturbance (Ding et al., 2012; Swenson et al., 2012). And our study also supports that three diversity metrics need to be simultaneously used to complement each other because three diversity metrics may have different patterns in ecosystems (Swenson et al., 2012; Purschke et al., 2013). Especially, we need to test phylogenetic signals in functional traits to assess phylogenetic relatedness as a proxy of functional similarity and if there were no signal, we have to use both metrics (Kraft and Ackerly, 2010; Yang et al., 2014; Chun and Lee, 2018).

However, despite of these strength and advantages of our studies, the present study has also limitations. First, this study investigated plant community structure and dynamics with few simply measurable traits like previous studies (Swenson et al., 2012; Purschke et al., 2013; Muscarella et al., 2016; Chun and Lee, 2018). Second, we only analyzed data for plants with stems ≥2 cm DBH, ignoring seedlings that may be more important in complex forest dynamics, and third, we analyzed data from four forest plots in geographically dispersed forests at different successional stages. Therefore, we suggest that future studies explore temporal changes in traits related to physiological and defense mechanisms that cannot be measured easily and are recognized to be important in community structuring and dynamics (Swenson et al., 2012). The study needs to include trees of all ages such as seedling, sapling and adult trees to give better understanding of temporal patterns of community-level change (Gross et al., 2015). And we need to increase the number of study sites and duration of long-term study to account for the generality in community structure and dynamics through whole forest successional stages (Norden et al., 2012; Swenson et al., 2012; Chai et al., 2016). Finally, it also needs to investigate the effects of environmental and demographic factors on diversity patterns to clarify and disentangle the role and relative importance of deterministic (environmental filtering and competitive exclusion) and neutral processes (Norden et al., 2012).

## Conclusions

A species-based approach to the analysis of changes in successional community assemblage is limited in the ability to reflect long-term evolutionary and functional trait responses of organisms to environment change (Swenson et al., 2012). Recent advances in the concept and analysis of community phylogenetics and functional traits have improved interpretations and understandings of community assembly processes (Webb et al., 2008; Kraft and Ackerly, 2010), and in this study, we quantified species, phylogenetic, and functional diversity of forest woody plant assemblages, through time. Our study emphasizes the complex and unpredictable interactions between community structure and the processes (Mayfield and Levine, 2010) and provides evidence of the different roles and importance of the different processes such as niche-based deterministic and neutral processes in forest plant communities. Although the role and relative importance of these two processes for the patterns of successional community structure differed among the study sites (Chase and Myers, 2011; Swenson et al., 2012; Chai et al., 2016), we found niche-based deterministic processes are the dominant drivers to structure plant community assembly during forest succession regardless of forest age and disturbance in this study. We tested for differences in temporal patterns of diversity at different forest strata and found temporal changes were different among the strata in the study sites through time. Therefore, our study suggests that forest strata need to be considered when studying plant community structures in forest ecosystems. Moreover, this study also revealed that the temporal changes of phylogenetic and functional diversity for understory strata in a forest (Mt. Geum), which were consecutively damaged by typhoon, were more extreme and larger than those of understory strata in the other sites. Recent researches also reported that plant community structures may differ with forest strata and such differences can be accelerated by disturbance (Ding et al., 2012; Swenson et al., 2012). And also this study supports that three diversity metrics need to be simultaneously used to complement each other because three diversity metrics may have different patterns in forest ecosystems (Swenson et al., 2012; Purschke et al., 2013). Especially, we need to test phylogenetic signal in functional traits to assess phylogenetic relatedness as a proxy of functional similarity and if there were no signal, we have to use both diversity metrics (Kraft and Ackerly, 2010; Yang et al., 2014; Chun and Lee, 2018). Our study suggests that contemporary forest ecosystems are composed of mosaics of plant communities that are formed by interactions among various processes. For a better understanding of plant community structure and dynamics and the processes during forest succession, we suggest further studies on traits associated with physiology and defense mechanisms, all life stages of woody plants, wider range of successional stages, and effects of environmental and/or demographic factors on diversity patterns.

## Author Contributions

J-HC and C-BL generated the key ideas and analyzed the data. J-HC designed and initiated the study, contributed to integration, visualization, and interpretation of the results, and contributed to the manuscript. C-BL co-designed and initiated the study, collated the plot data used in the analysis, contributed to the integration and interpretation of the data, and led the writing of the final manuscript. The authors have approved the final version to be published and agree to be accountable for all aspects of the work, ensuring questions related to its accuracy and integrity are appropriately investigated and resolved.

### Conflict of Interest Statement

The authors declare that the research was conducted in the absence of any commercial or financial relationships that could be construed as a potential conflict of interest.

## References

[B1] Blomberg S. P. Garland T. Jr. Ives A. R. (2003). Testing for phylogenetic signal in comparative data: behavioral traits are more labile. Evolution 57, 717–745. 10.1111/j.0014-3820.2003.tb00285.x 12778543

[B2] Chai Y. Yue M. Liu X. Guo Y. Wang M. Xu J.. (2016). Patterns of taxonomic, phylogenetic diversity during a long-term succession of forest on the Loess Plateau, China: insights into assembly process. Sci. Rep. 6:27087. 10.1038/srep27087 27272407 PMC4897607

[B3] Chase J. M. Myers J. A. (2011). Disentangling the importance of ecological niches from stochastic processed across scales. Philos. Trans. R. Soc. Lond. B Biol. Sci. 366, 2351–2363. 10.1098/rstb.2011.0063 21768151 PMC3130433

[B4] Cho Y. C. Shin H. C. Kim S. S. Lee C. S. (2007). Dynamics and conservation of the Gwangneung national forest in Central Korea: a national model for forest restoration. J. Plant Biol. 50, 615–625. 10.1007/BF03030604

[B5] Chun J. H. Lee C. B. (2018). Partitioning the regional and local drivers of phylogenetic and functional diversity along temperate elevational gradients on an East Asian peninsula. Sci. Rep. 8:2853. 10.1038/s41598-018-21266-4 29434300 PMC5809509

[B6] Chun J. H. Lim J. H. Kim S. H. Park C. R. Kwon T. S. Yang H. M. (2014). Long-Term Ecological Research on Forest Ecosystem Responses to Global Environmental Change. Seoul: National Institute of Forest Science.

[B7] Connell J. H. Slatyer R. O. (1977). Mechanisms of succession in natural communities and their role in community stability and organization. Am. Nat. 111, 1119–1144.

[B8] Conradi T. Temperton V. M. Kollmann J. (2017). Resource availability determines the importance of niche-based versus stochastic community assembly in grasslands. Oikos 126, 1134–1141. 10.1111/oik.03969

[B9] Ding Y. Zang R. Letcher S. G. Liu S. He F. (2012). Disturbance regime changes the trait distribution, phylogenetic structure and community assembly of tropical rain forests. Oikos 121, 1263–1270. 10.1111/j.1600-0706.2011.19992.x

[B10] Douma J. C. de Haan M. W. A. Aerts R. Witte J. P. M. van Bodegom P. M. (2012). Succession-induced trait shifts across a wide range of NW European ecosystems are driven by light and modulated by initial abiotic conditions. J. Ecol. 100, 366–380. 10.1111/j.1365-2745.2011.01932.x

[B11] Edwards E. J. Chatelet D. S. Sack L. Donoghue M. J. (2014). Leaf life span and the leaf economics spectrum in the context of whole plant architecture. J. Ecol. 102, 328–336. 10.1111/1365-2745.12209

[B12] Garnier E. Navas M. L. Grigulis K. (2016). Plant Functional Diversity: Organism Traits, Community Structure, and Ecosystem Properties. Oxford: Oxford University Press.

[B13] Gerhold P. Cahill J. F. Winter M. Bartish I. V. Prinzing A. (2015). Phylogenetic patterns are not proxies of community assembly mechanisms (they are far better). Funct. Ecol. 29, 600–614. 10.1111/1365-2435.12425

[B14] Gross I. Brandl R. Botzat A. Neuschulz E. L. Farwig N. (2015). Contrasting taxonomic and phylogenetic diversity responses to forest modifications: comparisons of taxa and successive plant life stages in South African scarp forest. PLoS ONE 10:e0118722 10.1371/journal.pone.0118722 25719204 PMC4342016

[B15] Helmus M. R. Keller W. B. Paterson M. J. Yan N. D. Cannon C. H. Rusak J. A. (2010). Communities contain closely related species during ecosystem disturbance. *Ecol*. Lett. 13, 162–174. 10.1111/j.1461-0248.2009.01411.x 20015255

[B16] Hwang T. Kang S. Kim J. Kim Y. Lee D. Band L. (2008). Evaluating drought effect on MODIS gross primary production (GPP) with an eco-hydrological model in the mountainous forest, East Asia. Glob. Chang. Biol. 14, 1037–1056. 10.1111/j.1365-2486.2008.01556.x

[B17] Johnson E. A. Miyanishi K. (2008). Testing the assumptions of chronosequences in succession. *Ecol*. Lett. 11, 419–431. 10.1111/j.1461-0248.2008.01173.x 18341585

[B18] Kim H. S. Park G. S. Lee S. M. Lee S. J. Lee H. G. Park H. W. (2016). A study of the vegetation structure of the geumsan in namhae-gun of Korea. Korean J. Environ. Ecol. 30, 214–227. 10.13047/KJEE.2016.30.2.214

[B19] Kraft N. J. B. Ackerly D. D. (2010). Functional trait and phylogenetic tests of community assembly across spatial scales in an Amazonian forest. Ecol. Monogr. 80, 401–422. 10.1890/09-1672.1

[B20] Laiju N. Otieno D. Jung E. Y. Lee B. Tenhunen J. Lim J. H. (2012). Environmental controls on growing-season sap flow density of *Quercus serrata* thunb in a temperate deciduous forest of Korea. *J. Ecol*. Environ. 35, 213–225. 10.5141/JEFB.2012.026

[B21] Lee S. M. Cho Y. C. Shin H. C. Oh W. S. Seol E. S. Park S. A. (2008). Successional changes in seed banks in abandoned rice fields in Gwangneung, Central Korea. *J. Ecol*. Environ. 31, 269–276. 10.5141/JEFB.2008.31.4.269

[B22] Letcher S. G. (2010). Phylogenetic structure of angiosperm communities during tropical forest succession. Proc. R. Soc. B 277, 97–104. 10.1098/rspb.2009.0865 19801375 PMC2842617

[B23] Letcher S. G. Chazdon R. L. (2009). Rapid recovery of biomass, species richness, and species composition in a forest chronosequence in northeastern Costa Rica. Biotropica 41, 608–617. 10.1111/j.1744-7429.2009.00517.x

[B24] Letten A. D. Keith D. A. Tozer M. G. (2014). Phylogenetic and functional dissimilarity does not increase during temporal heathland succession. Proc. R. Soc. B 281:20142102 10.1098/rspb.2014.2102 PMC4240997 25377459

[B25] Lim J. (1998). A Forest Dynamics Model Based on Topographically-Induced Heterogeneity in Solar Radiation and Soil Moisture on the Kwangneung Experimental Forest. Ph.D. thesis, Seoul: Seoul National University.

[B26] Lim J. H. Shin J. H. Jin G. Z. Chun J. H. Oh J. S. (2003). Forest stand structure, site characteristics and carbon budget of Gwangneung Natural Forest in Korea. Korean J. Agric. Forest Meteorol. 5, 101–109.

[B27] Loreau M. Naeem S. Inchausti P. Bengtsson J. Grime J. P. Hector A.. (2001). Biodiversity and ecosystem functioning: current knowledge and future challenges. Science 294, 804–808. 10.1126/science.1064088 11679658

[B28] Ma M. Zhou X. Qi W. Liu K. Jia P. Du G. (2013). Seasonal dynamics of the plant community and soil seed bank along a successional gradient in a subalpine meadow on the Tibetan Plateau. PLoS ONE 8:e80220. 10.1371/journal.pone.0080220 24244655 PMC3823802

[B29] Mason N. W. H. Richardson S. J. Peltzer D. A. de Bello F. Wardle D. A. Allen R. B. (2012). Changes in coexistence revealed by functional trait diversity. J. Ecol. 100, 678–689. 10.1111/j.1365-2745.2012.01965.x

[B30] Mayfield M. M. Levine J. M. (2010). Opposing effects of competitive exclusion on the phylogenetic structure of communities. Ecol. Lett. 13, 1085–1093. 10.1111/j.1461-0248.2010.01509.x 20576030

[B31] Moles A. T. Westoby M. (2006). Seed size and plant strategy across whole life cycle. Oikos 113, 91–105. 10.1111/j.0030-1299.2006.14194.x

[B32] Muscarella R. Uriarte M. Aide T. M. Erickson D. L. Forero-Montaña J. Kress W. J. (2016). Functional convergence and phylogenetic divergence during secondary succession of subtropical wet forests in Puerto Rico. J. Veg. Sci. 27, 283–294. 10.1111/jvs.12354

[B33] Myers J. A. Chase J. M. Jiménez I. Jørgensen P. M. Araujo-Murakami A. Paniagua-Zambrana N.. (2013). Beta-diversity in temperate and tropical forests reflects dissimilar mechanisms of community assembly. Ecol. Lett. 16, 151–157. 10.1111/ele.12021 23113954

[B34] Narwani A. Alexandrou M. A. Oakley T. H. Carroll I. T. Cardinale B. J. (2013). Experimental evidence that evolutionary relatedness does not affect the ecological mechanisms of coexistence in freshwater green algae. *Ecol*. Lett. 16, 1373–1381. 10.1111/ele.12182 24112458

[B35] Norden N. Letcher S. G. Boukili V. Swenson N. G. Chazdon R. (2012). Demographic drivers of successional changes in phylogenetic structure across life-history stages in plant communities. Ecology 93, S70–S82. 10.1890/10-2179.1

[B36] Pagel M. (1999). Inferring the historical patterns of biological evolution. Nature 401, 877–884. 10.1038/44766 10553904

[B37] Purschke O. Schmid B. C. Sykes M. T. Poschlod P. Michalski S. G. Durka W. (2013). Contrasting changes in taxonomic, phylogenetic and functional diversity during a long-term succession: insights into assembly processes. J. Ecol. 101, 857–866. 10.1111/1365-2745.12098

[B38] Qian H. Jin Y. (2016). An updated megaphylogeny of plants, a tool for generating plant phylogenies and an analysis of phylogenetic community structure. *J*. Plant Ecol. 9, 233–239. 10.1093/jpe/rtv047

[B39] Swenson N. G. Stegen J. C. Davies S. J. Erickson D. L. Forero-Montaña J. Hurlbert A. H.. (2012). Temporal turnover in the composition of tropical tree communities: functional determinism and phylogenetic stochasticity. Ecology 93, 490–499. 10.1890/11-1180.1 22624204

[B40] Walker L. R. Wardle D. A. Bardgett R. D. Clarkson B. D. (2010). The use of chronosequences in studies of ecological succession and soil development. J. Ecol. 98, 725–736. 10.1111/j.1365-2745.2010.01664.x

[B41] Webb C. O. Ackerly D. D. Kembel S. (2008). Phylocom: software for the analysis of phylogenetic community structure and trait evolution. Bioinformatics 24, 2099–2101. 10.1093/bioinformatics/btn358 18678590

[B42] Yang J. Zhang G. Ci X. Swenson N. G. Cao M. Sha L. (2014). Functional and phylogenetic assembly in a Chinese tropical tree community across size classes, spatial scales and habitats. Funct. Ecol. 28, 520–529. 10.1111/1365-2435.12176

[B43] Zanne A. E. Tank D. C. Cornwell W. K. Eastman J. M. Smith S. A. FitzJohn R. G.. (2014). Three keys to the radiation of angiosperms into freezing environments. Nature 506, 89–92. 10.1038/nature12872 24362564

