# Supplementary material for: Temporal Changes in Species, Phylogenetic, and Functional Diversity of Temperate Tree Communities: Insights From Assembly Patterns
Source: Front Plant Sci. 2019 Mar 19;10:294. doi: 10.3389/fpls.2019.00294 (PMC6433699; doi:10.3389/fpls.2019.00294)
Supplement: Supplementary file 2 [file Data_Sheet_2.PDF]

Supplementary Material

Temporal Changes in Species, Phylogenetic, and Functional Diversity of Temperate Tree Communities: Insights from Assembly Patterns

Jung-Hwa Chun[1], Chang-Bae Lee[2*]

[1]Reseach Planning and Coordination Division, National Institute of Forest Science, Seoul, Republic of Korea

[2]Department of Forestry, Environment and Systems, Kookmin University, Seoul, Republic of Korea

*** Correspondence:** Chang-Bae Lee, kecolee@kookmin.ac.kr

(A) Phylogenetic trees

Total for phylogenetic signal

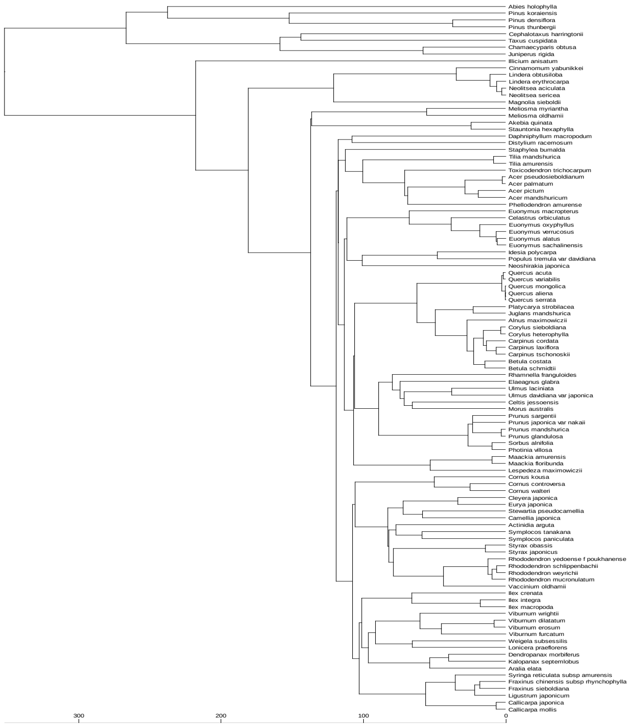

Gwangneung

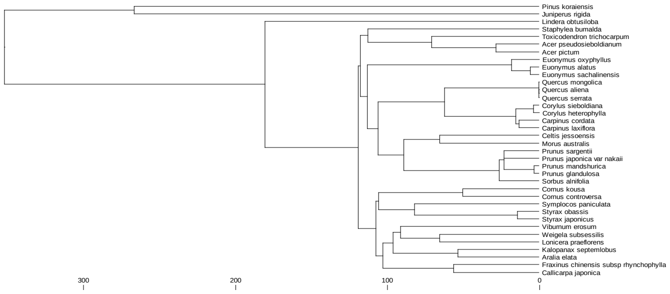

Mt. Gyebang

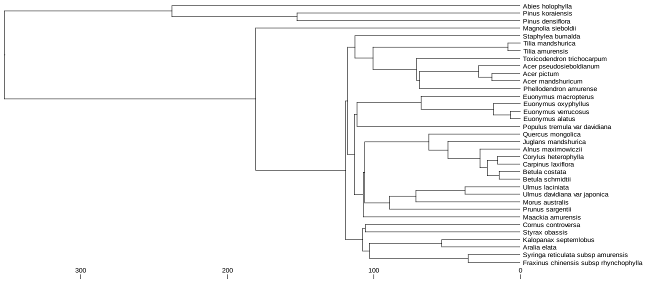

Mt. Geum

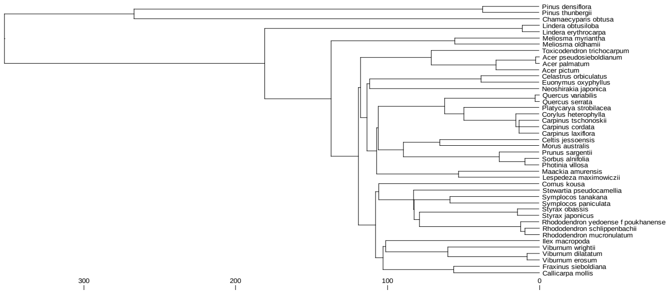

Mt. Halla

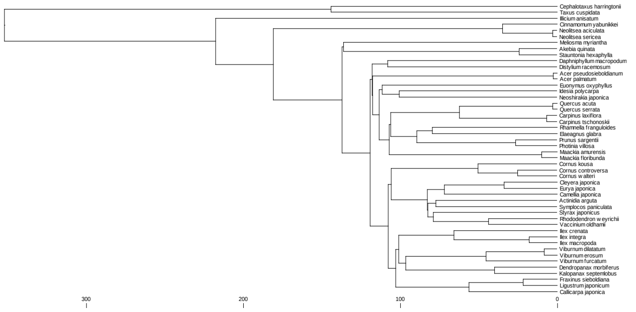

(B) Functional trait dendrograms

Gwangneung

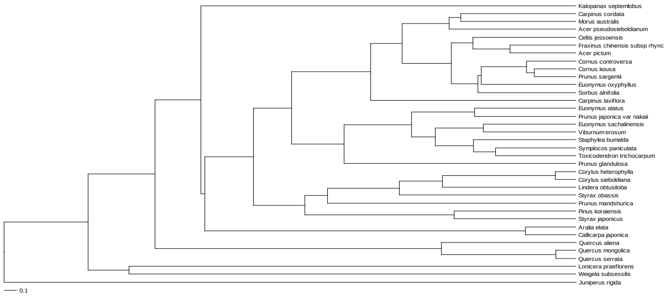

Mt. Gyebang

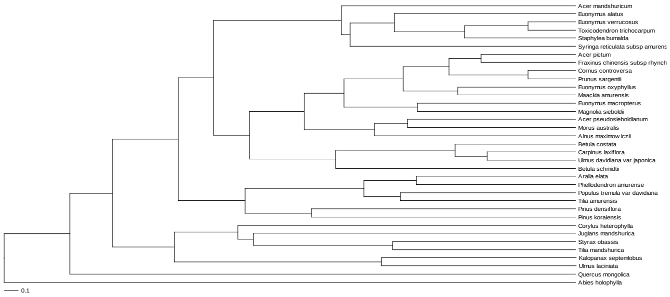

Mt. Geum

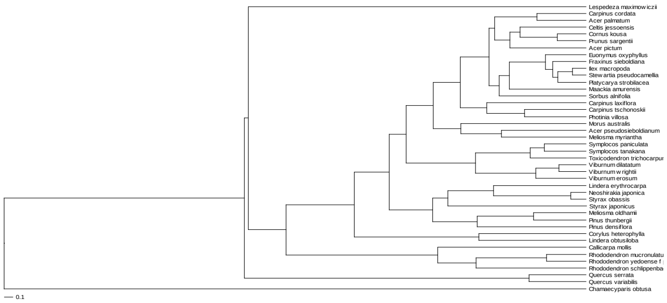

Mt. Halla

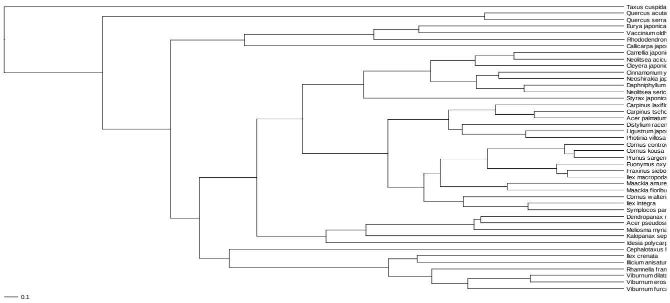

Supplementary Figure A1. (A) Phylogenetic trees and (B) functional trait dendrograms of wood species data.

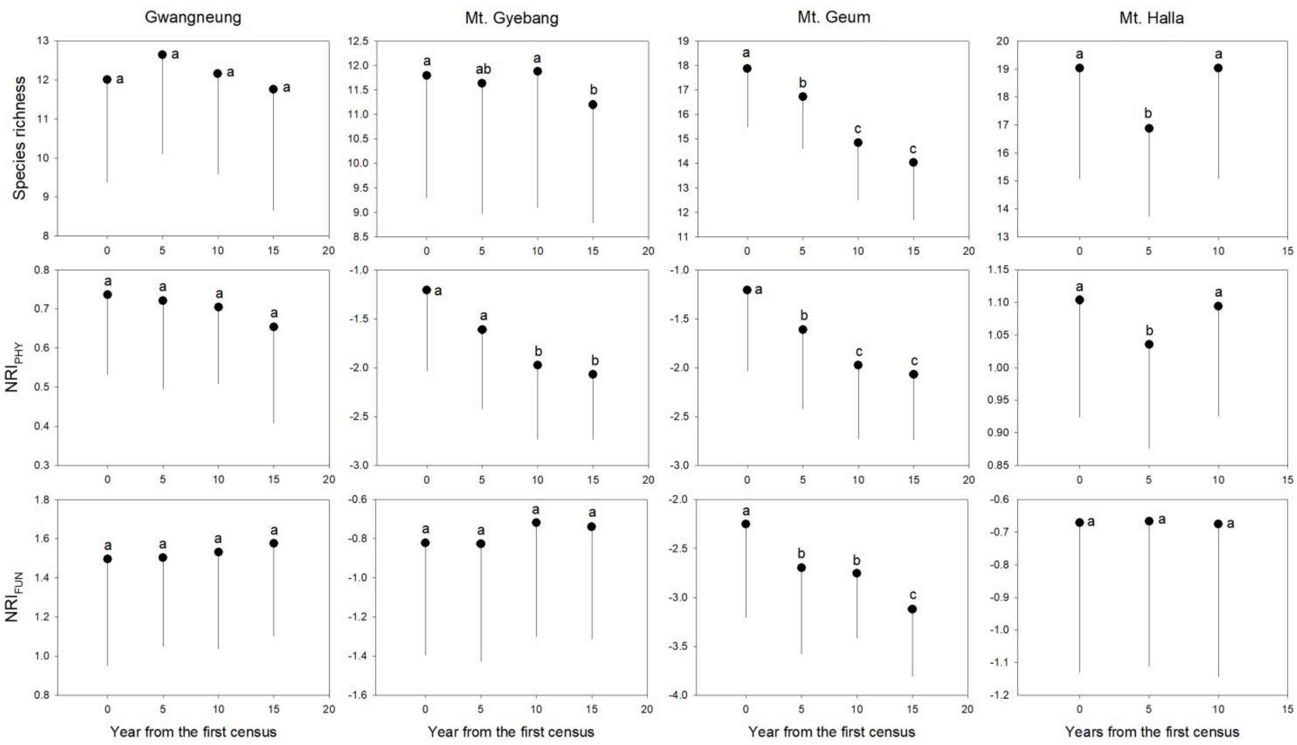

Supplementary Figure A2. Temporal changes in species, phylogenetic, and functional alpha diversity at whole strata. Letters represent significant differences ($P < 0.05$) between censuses. Mean values of each metric of diversity in each census of a study site significantly differed from zero using a one-sample t-test, indicating alpha diversity of the three diversity metrics was not random. Black lines indicate standard deviations. NRI_{PHY} and NRI_{FUN} represent phylogenetic and functional alpha diversity, respectively.

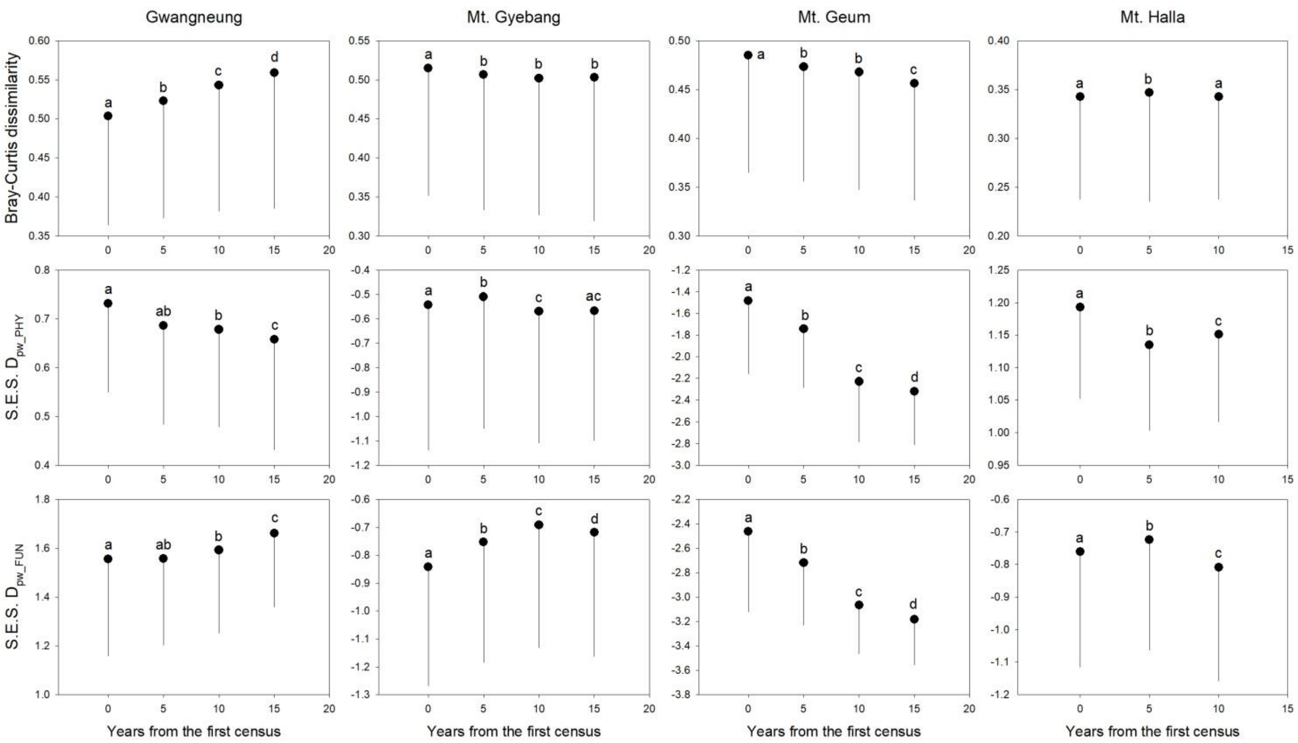

Supplementary Figure A3. Temporal changes in species, phylogenetic, and functional beta diversity between paired quadrats at whole strata. Letters represent significant differences (P < 0.05) between censuses. Mean values of each diversity metric in each census of a study site significantly differed from zero using a one-sample t-test, indicating beta diversity (turnover) of the three diversity metrics was not random. Black lines indicate standard deviations. S.E.S. D_{pw_PHY} and S.E.S. D_{pw_FUN} represent phylogenetic and functional beta diversity, respectively.

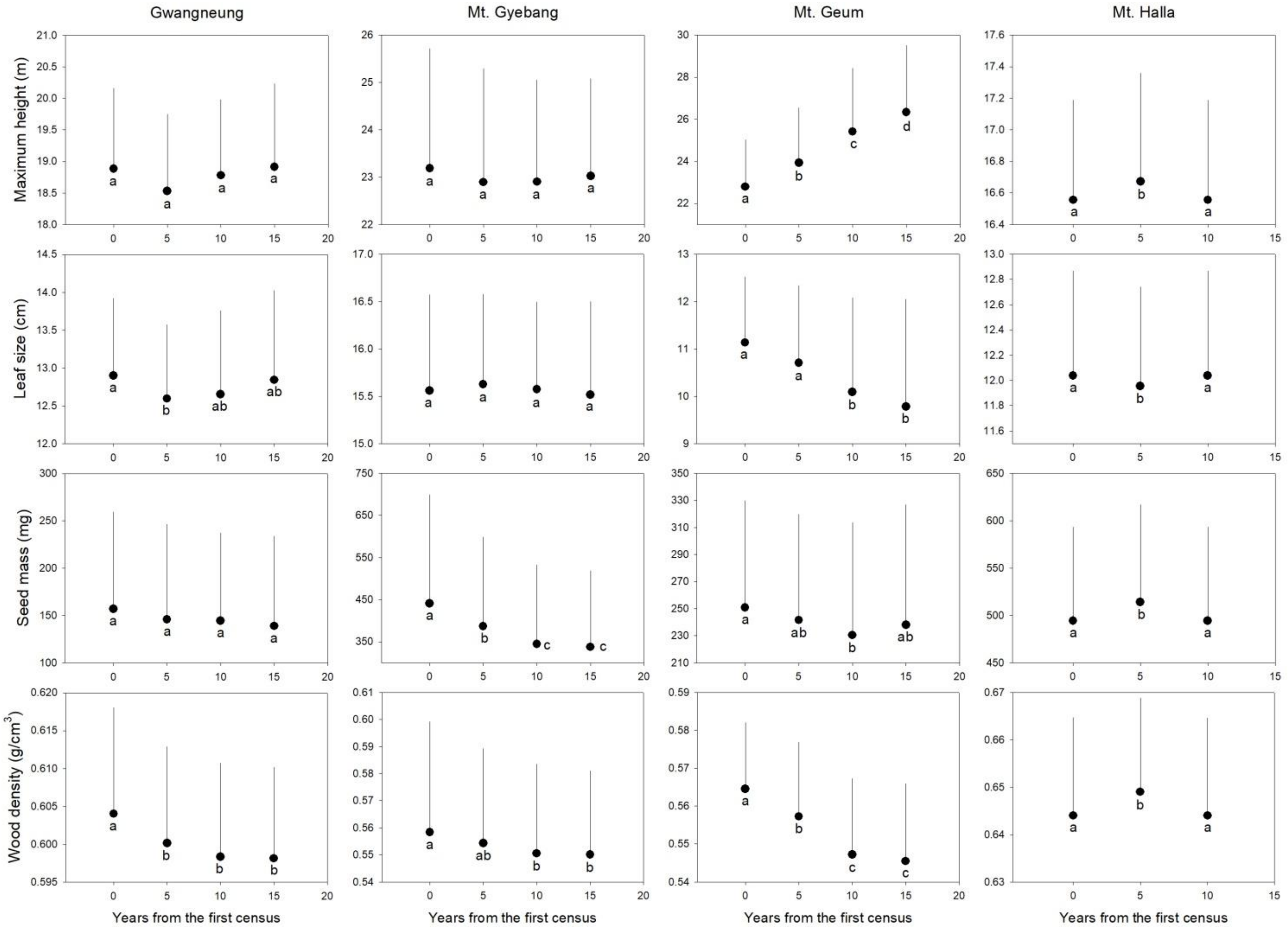

Supplementary Figure A4. Community-level mean values for the four functional traits through time at whole strata. Letters represent significant differences (P < 0.05) between censuses. Black lines indicate standard deviations.

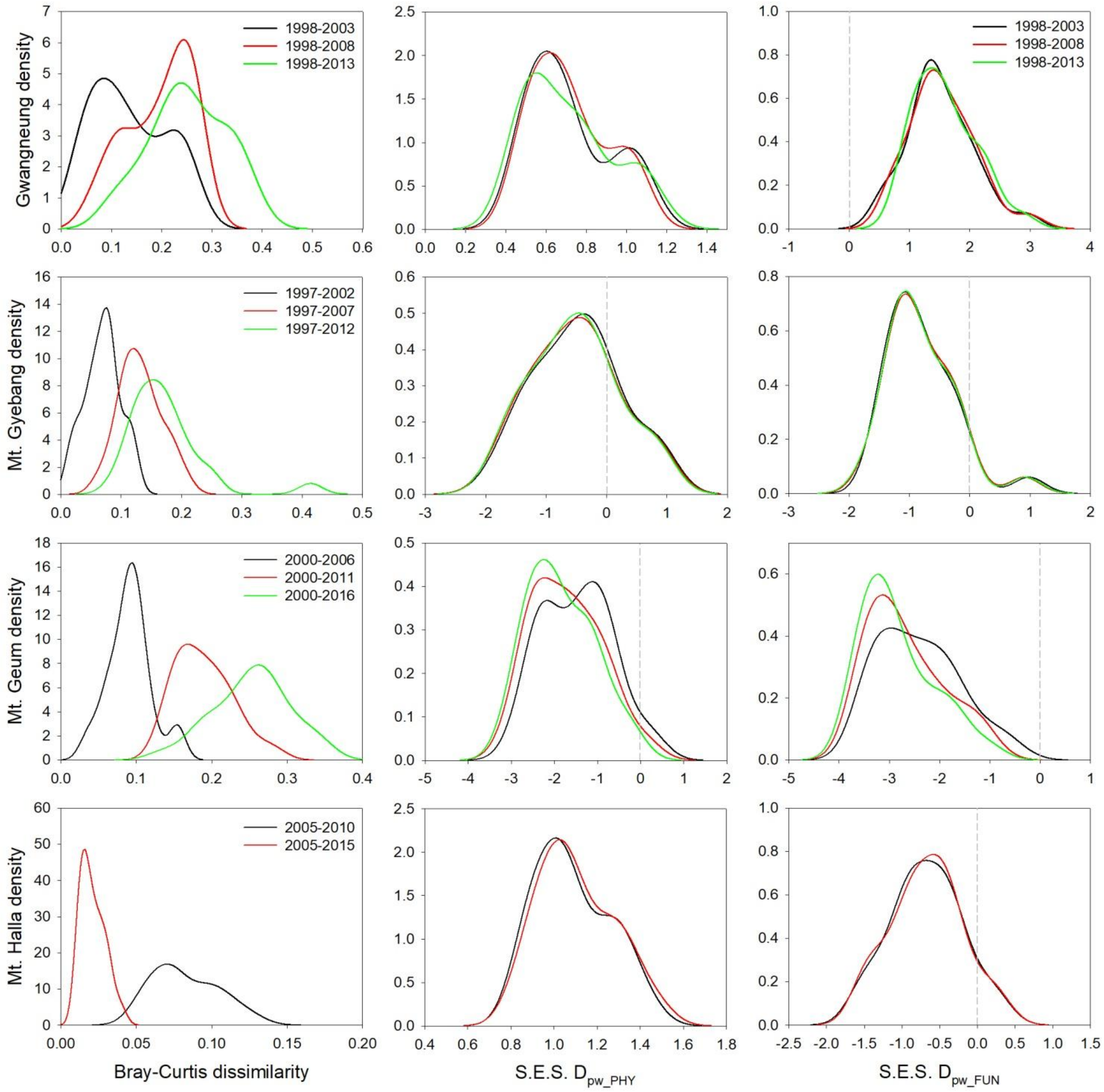

Supplementary Figure A5. Temporal turnover of species, phylogenetic and function composition between paired censuses in a quadrat quantified by Bray-Curtis dissimilarity for species turnover, S.E.S. D_{pw_PHY} and S.E.S. D_{pw_FUN} for phylogenetic and functional turnover, respectively. The plots were drawn by Kernel density estimation.
